# An N-Ethyl-N-Nitrosourea Induced Corticotropin-Releasing Hormone Promoter Mutation Provides a Mouse Model for Endogenous Glucocorticoid Excess

**DOI:** 10.1210/en.2013-1247

**Published:** 2013-12-03

**Authors:** Liz Bentley, Christopher T. Esapa, M. Andrew Nesbit, Rosie A. Head, Holly Evans, Darren Lath, Cheryl L. Scudamore, Tertius A. Hough, Christine Podrini, Fadil M. Hannan, William D. Fraser, Peter I. Croucher, Matthew A. Brown, Steve D. M. Brown, Roger D. Cox, Rajesh V. Thakker

**Affiliations:** Mammalian Genetics Unit (L.B., C.T.E., R.A.H., S.D.M.B., R.D.C.) and Mary Lyon Centre (C.L.S., T.A.H.), Medical Research Council Harwell, Harwell Science and Innovation Campus, Oxfordshire OX11 0RD, United Kingdom; Academic Endocrine Unit (C.T.E., M.A.N., R.A.H., F.M.H., R.V.T.), Nuffield Department of Clinical Medicine, University of Oxford, Oxford Centre for Diabetes, Endocrinology, and Metabolism, Churchill Hospital, Oxford OX3 7LE, United Kingdom; The Mellanby Centre for Bone Research (H.E., D.L.), Department of Human Metabolism, University of Sheffield, Sheffield S10 2RX, United Kingdom; Wellcome Trust Sanger Institute (C.P.), Wellcome Trust Genome Campus, Hinxton, Cambridge CB10 1SA, United Kingdom; Norwich Medical School (W.D.F.), University of East Anglia, Norwich Research Park, Norwich NR4 7TJ, United Kingdom; Garvan Institute of Medical Research (P.I.C.), Musculoskeletal Medicine Division, University of New South Wales, Sydney 2010, Australia; and University of Queensland Diamantina Institute (M.A.B.), Princess Alexandra Hospital, University of Queensland, Brisbane 4102, Australia

## Abstract

Cushing's syndrome, which is characterized by excessive circulating glucocorticoid concentrations, may be due to ACTH-dependent or -independent causes that include anterior pituitary and adrenal cortical tumors, respectively. ACTH secretion is stimulated by CRH, and we report a mouse model for Cushing's syndrome due to an N-ethyl-N-nitrosourea (ENU) induced *Crh* mutation at −120 bp of the promoter region, which significantly increased luciferase reporter activity and was thus a gain-of-function mutation. *Crh*^−*120*/+^ mice, when compared with wild-type littermates, had obesity, muscle wasting, thin skin, hair loss, and elevated plasma and urinary concentrations of corticosterone. In addition, *Crh*^−*120*/+^ mice had hyperglycemia, hyperfructosaminemia, hyperinsulinemia, hypercholesterolemia, hypertriglyceridemia, and hyperleptinemia but normal adiponectin. *Crh*^−*120*/+^ mice also had low bone mineral density, hypercalcemia, hypercalciuria, and decreased concentrations of plasma PTH and osteocalcin. Bone histomorphometry revealed *Crh*^−*120*/+^ mice to have significant reductions in mineralizing surface area, mineral apposition, bone formation rates, osteoblast number, and the percentage of corticoendosteal bone covered by osteoblasts, which was accompanied by an increase in adipocytes in the bone marrow. Thus, a mouse model for Cushing's syndrome has been established, and this will help in further elucidating the pathophysiological effects of glucocorticoid excess and in evaluating treatments for corticosteroid-induced osteoporosis.

Cushing's syndrome (CS), which is characterized by excessive circulating glucocorticoid (GC) concentrations, may be associated with obesity, redistribution of adipose tissue, diabetes mellitus, hypertension, muscle atrophy, subfertility, osteoporosis, and an increased susceptibility to infection (1). The etiology of CS can be broadly divided into ACTH-dependent or ACTH-independent causes. The ACTH-dependent forms are characterized by excessive ACTH production from a corticotroph adenoma, which is also referred to as pituitary-dependent CS or Cushing's disease; an ectopic tumor, also referred to as ectopic ACTH syndrome; or rarely from excessive hypothalamic secretion of CRH that may also arise from an ectopic tumor. ACTH-independent forms, apart from exogenous administration of GCs, result from GC hypersecretion by the adrenal cortex by mechanisms other than trophic ACTH stimulation. These ACTH-independent causes include the following: unilateral adrenal cortical tumors, which may be adenomas or carcinomas; and bilateral adrenal abnormalities, which may be due to primary pigmented nodular adrenal disease associated with the Carney complex (CNC), McCune-Albright syndrome (MAS), or macronodular disease related to aberrations of the cAMP signaling pathway and ectopic expression of G-protein coupled receptors (2).

CS may also occur as an isolated familial endocrinopathy or as part of a complex syndromic disorder such as CNC, MAS, and multiple endocrine neoplasia type 1 (MEN1), and studies of these forms have helped to elucidate the underlying genetic abnormalities. Thus, two patients with isolated familial CS due to ACTH-secreting adenomas have been reported with mutations in the aryl hydrocarbon receptor-interacting protein (*AIP*) (3, 4); less than 10 MEN1 patients with CS have been reported to have mutations of the tumor suppressor *MEN1*, which encodes menin, a protein that has a role in transcription regulation, cell proliferation, and genome stability (4–8); more than 80% of the 70% of CNC patients who have CS have been observed to have mutations in the protein kinase A regulatory subunit 1α (*PRKAR1A*), a mediator involved in the inhibition of cAMP signaling; and less than 10% of MAS patients who have CS may have somatic-activating mutations in the imprinted α-subunit of the stimulatory guanine nucleotide binding protein G_s_α (9–11). The precise cellular and physiological mechanisms that cause CS resulting from these different etiologies still remain to be elucidated, although such studies have been greatly helped by the availability of mouse models for CS. These CS models include those generated by chronic ACTH infusion (12); CRH overexpression by transgenesis (13); deletion, by homologous recombination, of neuroendocrine protein 7B2, resulting in ACTH hypersecretion (14); and adrenal cortex-specific deletion of the CNC-associated gene *Prkar1a* (15). All of these mouse models develop some features of CS, although there are some notable differences; however, the effects of endogenous glucocorticoid excess on bone have only recently been reported in the CRH-overexpressing transgenic model (16). Here we report on the bone, glucose, and adipose abnormalities in a mouse model with the features of CS, identified during the course of our phenotypic-driven studies of progeny from mice mutagenized with the chemical N-ethyl-N-nitrosourea (ENU) that introduces point mutations that may result in partial loss of function, gain of function, and null alleles (17).

## Materials and Methods

### ENU mutagenesis

All animal studies were carried out using guidelines issued by the UK Medical Research Council, in Responsibility in Use of Animals for Medical Research (July 1993) and Home Office Project License numbers 30/2433 and 30/2642. ENU-treated G0 C57BL/6J male mice were mated to C3H/HeH female mice to produce G1 progeny, which were screened for phenotypes (17).

### Mapping and sequencing

Genomic DNA was extracted from tail or auricular biopsies using the DNeasy kit (QIAGEN). For genome-wide mapping, genomic DNA was genotyped by KBioscience (www.kbioscience.co.uk, now known as LGC Genomics, www.lgcgenomics.com) using a panel of 91 single-nucleotide polymorphic (SNP) markers arranged in chromosome sets. The individual exons and the promoter region of *Crh* were amplified from genomic DNA by PCR using gene-specific primers and AmpliTaq Gold PCR master mix (Life Technologies) and the PCR products sequenced by Source Bioscience using the Sanger sequencing service (www.lifesciences.sourcebioscience.com).

### Histological analysis

Pituitary and adrenal tissues were dissected and fixed in neutral buffered formalin, and 5-μm paraffin sections were stained with hematoxylin and eosin (H&E).

### Analysis of growth and body composition

To determine body composition and body weight, mice were subjected to quantitative nuclear magnetic resonance (EchoMRI; www.echomri.com) and weighed weekly from weaning.

### Plasma biochemistry and hormone analysis

Blood samples were collected from the lateral tail vein after the application of topical local anesthesia and while mice were being restrained in a Perspex bleeding tube. Blood collection was also undertaken by cardiac puncture after terminal anesthesia. Blood was sampled for corticosterone measurement between 11:30 and 12:30 pm (4.5–5.5 h after lights on) for female and between 1:00 and 2:00 pm (6–7 h after lights on) for male CS mice and wild-type (WT) littermate controls. To evaluate the corticosterone circadian rhythm, plasma samples for corticosterone measurements were collected at the circadian nadir (morning, 1 h after lights on) and circadian peak (evening, 1 h before lights out). Blood was sampled for ACTH measurement between 12:00 and 2:00 pm (5–7 h after lights on) for both CS mice and WT littermates.

Plasma was separated by centrifugation at 800 × *g* for 10 minutes at 4°C and stored at −20°C prior to analysis. Plasma samples were analyzed for sodium, potassium, chloride, total calcium, inorganic phosphate, urea, creatinine, albumin, fructosamine, glucose, uric acid, total protein, total cholesterol, triglycerides, alkaline phosphatase (ALP), alanine aminotransferase (ALT), and aspartate aminotransferase (AST) on a Beckman Coulter AU400 analyzer, as described previously (18, 19). Plasma calcium was adjusted for variations in albumin concentrations using the following formula: [plasma calcium (millimoles per liter)] − [(plasma albumin (grams per liter) − 30) × 0.017]. Plasma concentrations of corticosterone were quantified using an AssayMax ELISA kit (AssayPro). Hormones were measured as follows: PTH using a two-site ELISA kit (Immunotopics); insulin, glucagon, and leptin using a mouse endocrine multiplex immunoassay kit (Millipore); ACTH was quantified using the mouse bone panel kit (Millipore); and osteocalcin and total adiponectin using mouse singleplex immunoassay kits (Millipore). Details of the Millipore assays performance characteristics are provided in Supplemental Materials and Methods, published on The Endocrine Society's Journals Online web site at http://endo.endojournals.org.

### Metabolic cages and urine biochemistry analysis

Twelve-week-old mice were individually housed in metabolic cages (Techniplast) for 24 hours, as described (20). Animal weight, 24-hour water and food consumption, and 24-hour urine volume were recorded. Prior to analysis, all measurements of food/water intake and urine volume were expressed as values per 100 g of body weight. Urine samples were taken for analysis; each sample was centrifuged at 800 × *g* for 10 minutes at 4°C and aliquots stored at −20°C prior to analysis. Diluted (1:4 with distilled water) and undiluted samples were analyzed for sodium, potassium, chloride, urea, inorganic phosphate, glucose, total protein, and creatinine using an Olympus AU400 analyzer, as described previously (18). The concentrations of urinary glucose and calcium in millimoles per liter were expressed as a ratio to the concentration of urinary creatinine in millimoles per liter. To evaluate the corticosterone circadian rhythm, urine samples for corticosterone measurements were collected at the circadian nadir (morning, 1 h after lights on) and circadian peak (evening, 1 h before lights out). Urinary concentrations of corticosterone were quantified using an AssayMax ELISA kit (AssayPro).

### Luciferase reporter assays

Neuro2a cells were transiently transfected with a total of 300 ng of plasmid DNA per well using GeneJammer transfection reagent (Stratagene) as follows: 200 ng/well of pGL4 firefly luciferase reporter plasmid containing either the WT or mutant Crh promoter −501 to +1, or empty pGL4 vector and 100 ng/well of plasmid encoding Renilla luciferase (pRL-null Vector; Promega) to allow normalization of the data. Cells were harvested 48 hours after transfection and assayed for luciferase activity using the dual luciferase reporter assay (Promega). Three experiments were carried out in triplicate, and data are presented as mean firefly to renilla ratio ± SEM of all experiments.

### Real-time PCR analysis

Total RNA was isolated from pituitary samples using the RNAeasy minikit (QIAGEN). Reverse transcription was done using Superscript III reverse transcriptase (Invitrogen). Quantitative PCR was performed using a 7500 Fast real-time PCR system using TaqMan gene expression assays (Applied Biosystems) for *Pomc* (Mm00435874_m1) and *Gapdh* (Mm99999915_g1).

### X-ray and dual-energy X-ray absorptiometry (DEXA) imaging

Anesthetized mice were subjected to digital X-ray analysis using a Faxitron MX-20 radiography system (Faxitron X-Ray LLC) and DEXA using a Lunar PIXImus densitometer (GE Medical Systems), and the DEXA images were processed using the PIXImus software (GE Medical Systems).

### Dynamic histomorphometry

Tibiae from 12-week-old male and female *Crh*^−*120*/+^ and WT calcein-injected mice were fixed in 70% ethanol, processed, and embedded in LRWhite medium resin. Two levels (50 μm apart) of 10-μm sections were examined under UV illumination using a DMRB light microscope (Leica Microsystems). The proportion of corticoendosteal bone undergoing mineralization and the separation between the two fluorescent labels was measured using the Osteomeasure system. For corticoendosteal bone analysis, 12 250-μm × 250-μm fields were analyzed for a total of 3 mm length, starting at 250 μm from the growth plate. The percentage mineralized surface was calculated as [(dL + 0.5 sL)/BPm.] × 100, where dL is the double-label perimeter, sL is the single-label perimeter, and BPm. is the total bone perimeter. The mineral apposition rate (MAR; micrometers per day) was calculated as interlabel width/number of days between injections. The bone formation rate (square millimeters ×10^−3^/mm/d) was calculated as follows: [MAR × (dL + 0.5 sL)]/BPm. A 0.75-mm^2^ area, 250 μm from the growth plate, was analyzed to determine the number of osteoblasts per millimeter of bone, percentage of bone covered by osteoblasts, and adipocyte number.

### Statistical analysis

Data are presented as group mean ± SEM. Statistical comparisons were performed between age- and sex-matched groups using a two-tailed, unpaired Student's *t* test. An unpaired *t* test with Welch's correction, Mann-Whitney test, Fisher's exact test, and one-way ANOVA with repeated measures and Bonferroni posttest were performed using GraphPad Prism version 5.04 for Windows (GraphPad Software, www.graphpad.com. A value of *P* ≤ .05 was considered to be statistically significant.

## Results

### Identification of an ENU-induced mouse model with obesity, diabetes mellitus, and reduced bone mineral density (BMD) consistent with CS

**Figure 1.**
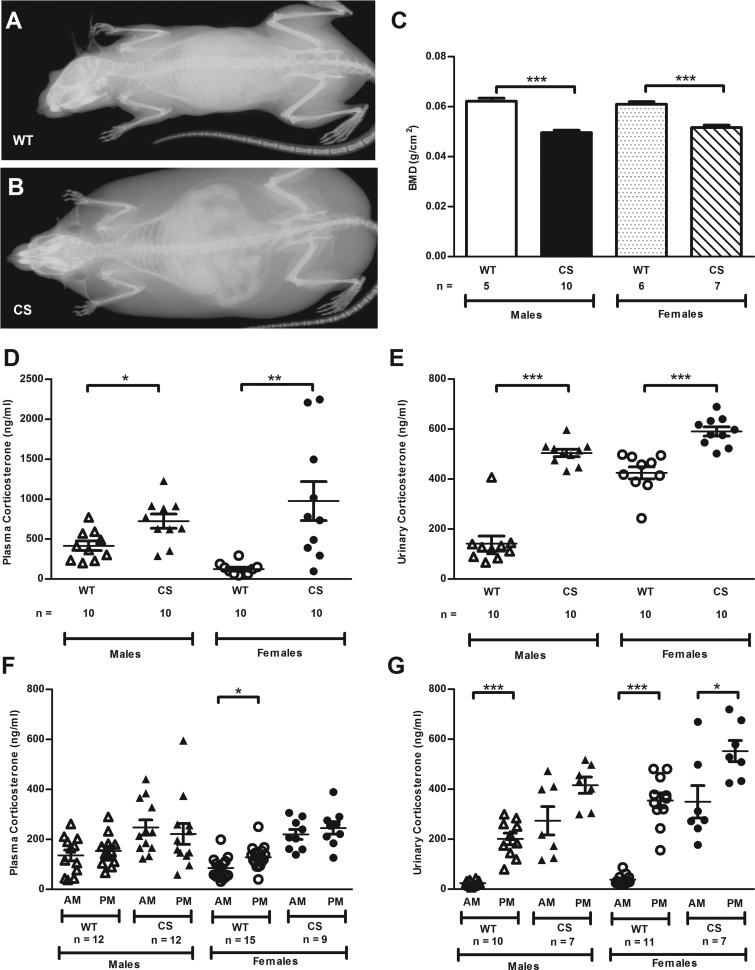
CS model phenotype. A and B, Radiographs of 12-week-old female WT littermate (A) and CS mouse (B). C, Whole-body BMD assessed by DEXA analysis of 12-week-old WT and CS mice. Plasma corticosterone (D) and urinary corticosterone concentrations (E) in free-fed WT and CS mice on a C3H/HeH (93.75%) background at 9 weeks of age. Plasma corticosterone (F) and urinary corticosterone (G) concentrations in free-fed, 9-week-old WT and CS mice on a congenic C57BL/6J background at the circadian nadir (AM) and circadian peak (PM). Mean ± SEM are shown and *P* values were calculated between age- and sex-matched groups using an unpaired *t* test with Welch's correction. *, *P* < .05; **, *P* < .01; ***, *P* < .001.

Progeny of ENU-mutagenized C57BL/6J male mice were phenotypically assessed for metabolic diseases, including diabetes mellitus, obesity, and bone and mineral disorders. This identified a founder G1 female that was obese (39.8 g; population mean ± SD 29 ± 3.3 g) and hyperglycemic [14.8 mmol/L (266.7 mg/dL); population mean ± SD 11.8 ± 1.1 mmol/L (212.6 ± 19.8 mg/dL)]. Breeding of this mouse, by crossing to C3H/HeH, for inheritance testing yielded 32 progeny (16 males and 16 females) of whom 7 of 16 males and 9 of 16 females, that is, 50% had inherited obesity and hyperglycemia, consistent with an autosomal dominant trait. X-ray (Figure 1, A and B) and DEXA analysis of additional progeny revealed that 17 mice (10 males and seven females) affected with obesity and hyperglycemia also had a significant reduction (*P* < .001) in BMD (Figure 1C). The affected mice also had thinner tails, premature hair loss, and thin skin. This combination of obesity, hyperglycemia, and low BMD is consistent with the occurrence of CS; therefore, plasma and urinary corticosterone measurements were undertaken in 9-week-old mice (Figure 1, D and E). This revealed the CS male and female mice to have significantly elevated plasma (*P* < .05) and urinary (*P* < .001) corticosterone concentrations (Figure 1, D and E). Moreover, an analysis of the circadian nadir and peak urinary corticosterone concentrations revealed a diminution of the corticosterone circadian rhythm in CS mice compared with WT littermates (Figure 1, F and G). Thus, male and female WT mice displayed a peak urinary corticosterone concentration that was 8-fold greater than the nadir value (*P* < .001), whereas the corresponding mean fold change of male and female CS mice was much reduced (1.5-fold, males *P* = ns and females *P* < .05) (Supplemental Figure 1). The diurnal plasma corticosterone results are somewhat confounded by the effects of stress in blood collection (Supplemental Figure 1). These biochemical findings established the diagnosis of CS in affected mice.

**Figure 2.**
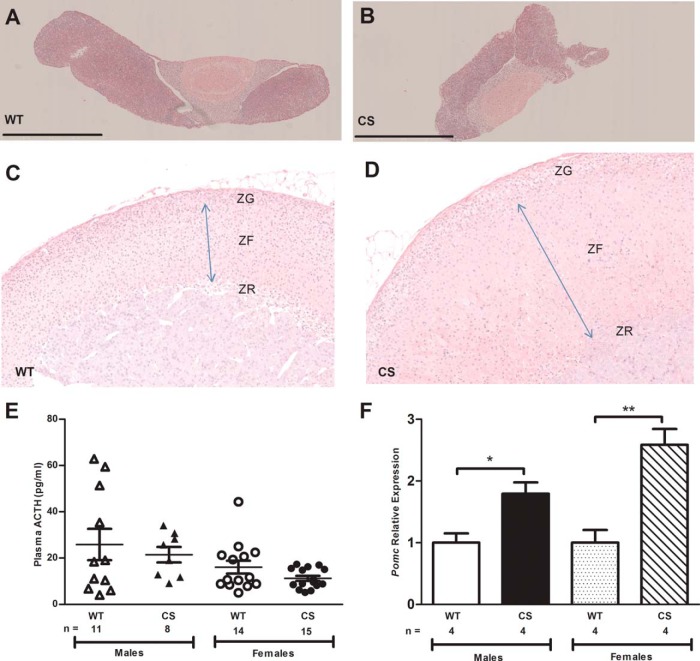
Assessment of pituitary-adrenal axis. A and B, H&E-stained sections of pituitaries from 17-week-old WT (A) and CS (B) mice. Scale bars, 1 mm. C and D, H&E-stained sections of adrenals from 17-week-old WT (C) and CS (D) mice. Images taken at the same magnification showing hypertrophy of zona fasiculata (ZF), indicated by double-headed arrow, zona glomerulosa (ZG), and zona reticularis (ZR). E, Plasma ACTH concentrations in free-fed WT and CS mice at 17 weeks of age. F, Quantitative PCR analysis of *Pomc* in pituitaries of WT and CS mice at 14 weeks of age (males) and 22 weeks of age (females). Mean ± SEM are shown, and *P* values were calculated between age- and sex-matched groups using an unpaired *t* test with Welch's correction. *, *P* < .05; **, *P* < .01.

To further establish the diagnosis and etiology of CS in the mutant mice, the pituitary-adrenal axis was investigated. Histological analysis of the anterior pituitary (Figure 2, A and B) and adrenal glands (Figure 2, C and D) did not reveal the occurrence of any tumors or other abnormalities associated with CS, although the adrenal glands of the CS mice were heavier than those of their WT littermates (adrenal weights normalized to body weight, mean ± SEM: male WT = 0.19 ± 0.02 mg/g vs male CS = 0.37 ± 0.02 mg/g, *P* < .001; female WT = 0.29 ± 0.09 mg/g vs female CS = 0.62 ± 0.05 mg/g, *P* < .05), which corresponded with hypertrophy of the zona fasciulata (Figure 2D). No change was noted in the zona glomerulosa (Figure 2D). Excess ACTH stimulates growth of the zona fasciculata of the adrenal cortex, thereby resulting in hyperplasia and increased adrenal weight. However, plasma ACTH concentrations were similar in WT and CS mice (Figure 2E), thereby indicating that anterior pituitary adenomas or ectopic ACTH production were unlikely causes of CS in these mice. The inappropriately normal plasma ACTH concentrations in the presence of high corticosterone concentrations in the CS mice suggests that the pituitary corticotrophs may be stimulated by CRH to produce ACTH, and the finding of a significant increase in expression of promelanocorticotropin (POMC) detected by quantitative PCR (Figure 2F) in the anterior pituitaries of CS males and females would be consistent with this notion. Thus, these data established that the CS mice had hypercorticosteronemia, which was not due to an adrenal or anterior pituitary tumor but may possibly instead be due to overstimulation of the pituitary corticotrophs from the hypothalamus, which secretes CRH.

### Mapping of the CS locus to chromosome 3 and identification of a gain-of-function mutation in the promoter of the *Crh* gene

**Figure 3.**
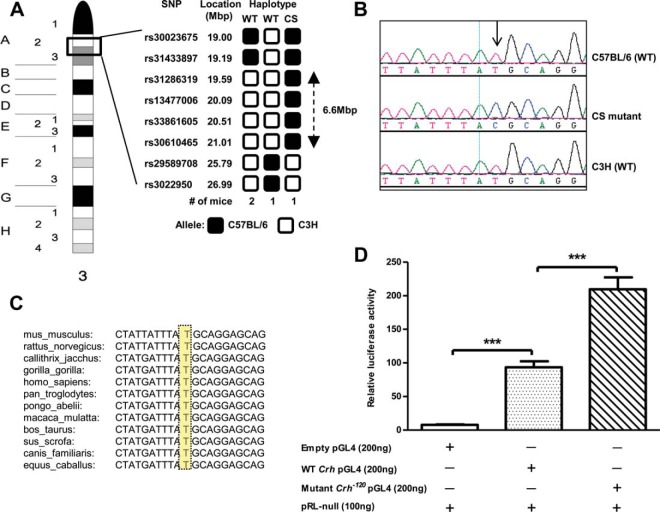
Mapping and identification of the *Crh*^−*120*^ mutation. A, Mapping and haplotype analysis located the CS locus to a 6.6-Mbp region between rs31433897 and rs29589708 on chromosome 3. The CS locus is inherited with C57BL/6 alleles and the haplotypes obtained from WT and mutant CS mice are shown separately. The 6.6-Mbp interval contained 22 genes, which included that for *Crh*. B, DNA sequence analysis of the *Crh* gene identified a T-to-C transition (arrow) in the promoter region at −120 bp upstream of the start codon. C, Sequence alignment of the *Crh* gene promoter region around −120 bp upstream of the start codon in 12 eutherian mammals using ENSEMBL (59) shows the T nucleotide at −120 (highlighted in yellow) to be conserved. D, Relative luciferase activity in Neuro2a cells that were cotransfected with WT *Crh* and mutant *Crh*^−*120*^ promoter pGL4 constructs or empty pGL4 vector and plasmid encoding Renilla luciferase (pRL-null). Mean ± SEM are shown (n = 18); *P* values were calculated using a Student's *t* test. ***, *P* < .001. +, Presence of vector; −, absence of vector.

Genome-wide mapping studies using 91 SNPs and DNA samples from 55 mice (23 affected and 32 unaffected) mapped the CS locus to chromosome 3 (Figure 3A). CS was found to cosegregate with C57BL/6J alleles identified by five SNPs from chromosome 3 and an examination of the haplotypes, which revealed recombinants, further localized the CS locus to a 6.6-Mb interval flanked by rs31433897 and rs29589708 on proximal mouse chromosome 3 (Figure 3A). This interval could not be refined further because strain-specific polymorphic loci within this region are not available. This interval contains 22 genes, which includes the *Crh* gene, and because the phenotypic data (Figure 2E) had revealed inappropriate ACTH secretion by likely stimulation of pituitary corticotrophs, we decided to investigate the *Crh* gene for mutations. DNA sequence analysis of the two exons and two exon-intron boundaries of the *Crh* gene (ENSMUSG00000049796) did not identify any abnormalities. Moreover, DNA sequence analysis of the other 21 genes in the interval also did not identify any abnormalities. However, DNA sequence analysis of the 2.4-kb region upstream of the *Crh* coding region, which contains the *Crh* promoter and 5′ untranslated region, identified a T-to-C transition at −120 bp (Figure 3B) with reference to a transcription start (NM_205769) (21). The *Crh*^−*120*^ abnormality was demonstrated to cosegregate with CS by use of a pyrosequencing assay (Supplemental Figure 2) in 55 progeny (23 affected and 32 unaffected). Moreover, the T nucleotide at −120 bp is evolutionarily conserved (Figure 3C) and occurs within the consensus protein sequence of a caudal-type homeobox response element (CDXARE), which is also highly conserved across species.

CDXARE is a cAMP response element that has been reported to have a key role in the regulation of *Crh* gene expression (22, 23). To assess the effects of the *Crh*^−*120*^ mutation on gene expression, we used a luciferase reporter assay (Figure 3D), in which WT *Crh* and mutant *Crh*^−*120*^ promoter pGL4 reporter constructs were transfected into Neuro2a cells, which have previously been used to investigate regulation of *Crh* promoter activity (24). Luciferase expression from the WT *Crh* promoter resulted in a significant increase, by 12-fold in relative luciferase activity, when compared with that of the empty pGL4 luciferase vector. Luciferase expression from the *Crh*^−*120*^ mutant promoter resulted in a 27-fold increase in luciferase activity when compared with the empty pGL4 luciferase vector and a significant increase, by 2-fold in relative luciferase activity, when compared with the WT *Crh* promoter, thereby demonstrating that the mutation causes an increase in basal gene transcription. The increase of expression by 2-fold in luciferase activity from the *Crh*^−*120*^ mutant promoter is consistent with the observed in vivo effects of the increased expression of CRH, namely increased POMC expression (Figure 2F) and inappropriately normal plasma ACTH concentrations (Figure 2E) in the presence of hypercorticosteronemia (Figure 1D).

### Studies of fat and lean mass and glucose and lipid metabolism

**Figure 4.**
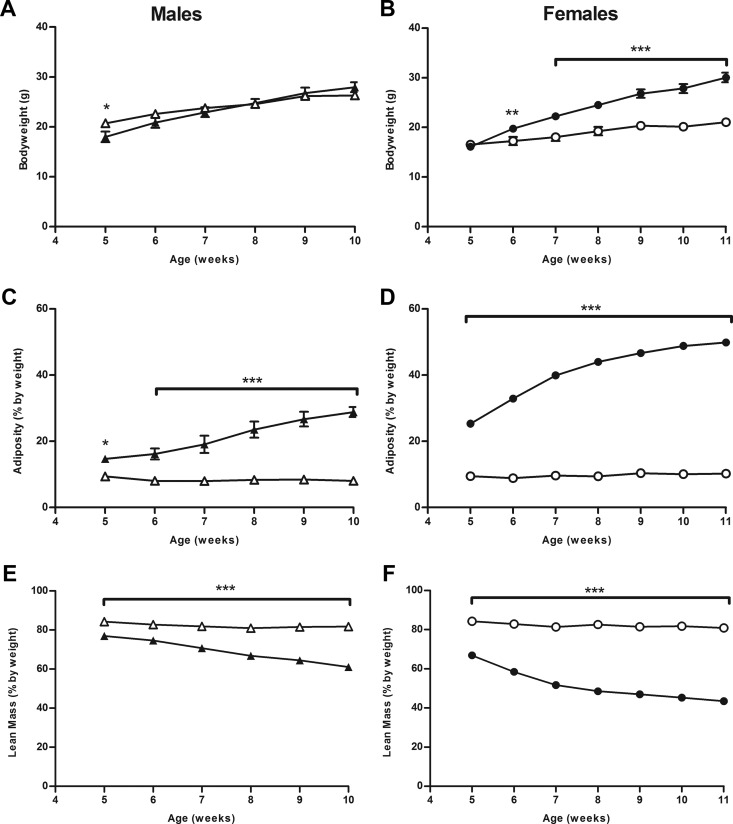
Fat deposition is increased in *Crh*^−*120*/+^ mice. A and B, Body weights of male *Crh*^−*120*/+^ (A) and female *Crh*^−*120*/+^ (B) mice compared with WT littermates on a C57BL/6J (98.4%) background. C and D, Adiposity, as measured by EchoMRI, of male (C) and female (D) *Crh*^−*120*/+^ mice compared with WT littermates. E and F, Lean mass, as measured by EchoMRI, of male (E) and female (F) *Crh*^−*120*/+^ mice compared with WT littermates. Males and females are shown by triangles and circles, respectively; WT and *Crh*^−*120*/+^ mice are shown by open and filled symbols, respectively. A–F, Vertical bars are mean ± SEM with n = 5 for all groups at all points. C, D, and F, SEM values that were less than 1.1% are not shown. E, SEM values that were less than 1.9% are not shown. *P* values were calculated between age- and sex-matched groups using an unpaired *t* test with Welch's correction and repeated-measures ANOVA with Bonferroni posttest. *, *P* < .05; **, *P* < .01; ***, *P* < .001.

**Figure 5.**
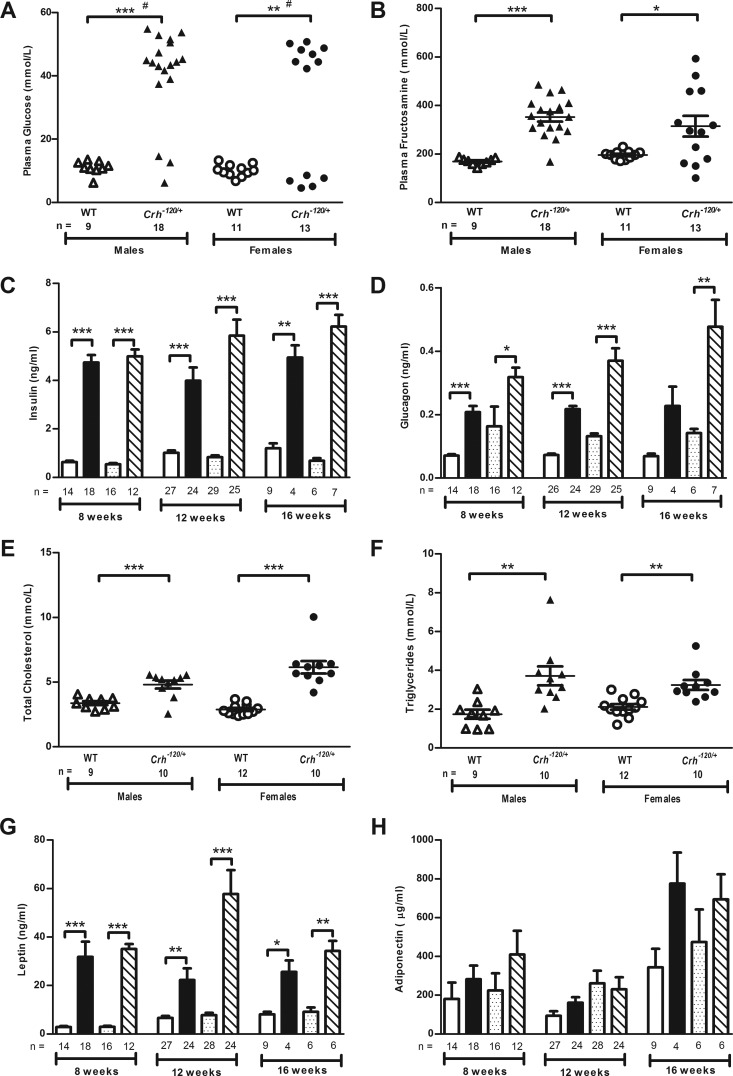
Dysregulation of glucose and lipid metabolism in *Crh*^−*120*/+^ mice. Plasma glucose (A) and plasma fructosamine (B) concentrations in free-fed WT and *Crh*^−*120*/+^ mice between 12 and 36 weeks of age. Plasma insulin (C) and plasma glucagon (D) concentrations after a 4-hour fast in WT and *Crh*^−*120*/+^ mice at several time points. Plasma cholesterol (E) and plasma triglyceride (F) concentrations in 4-hour-fasted WT and *Crh*^−*120*/+^ mice between 23 and 27 weeks of age. Plasma leptin (G) and plasma adiponectin (H) concentrations after a 4-hour fast in WT and *Crh*^−*120*/+^ mice at several time points. Histograms show WT males (unshaded), *Crh*^−*120*/+^ males (black), WT females (dotted), and *Crh*^−*120*/+^ females (hatched). Results are mean ± SEM. *P* values were calculated between age- and sex-matched groups using an unpaired *t* test with Welch's correction apart from (A). #, Data analyzed using Fisher's exact test; *, *P* < .05; **, *P* < .01; ***, *P* < .001.

The *Crh*^−*120*^ mutation had significant effects on body weight, adiposity, and lean mass (Figure 4) in males and females, although a gender difference was observed in body weight. Thus, male heterozygous *Crh*^−*120*/+^ mice were significantly lighter at 5 weeks of age, but there was no significant difference in body weight from 6 weeks of age on a C57BL/6J background (Figure 4A). However, female heterozygous *Crh*^−*120*/+^ mice were significantly heavier from 6 weeks of age on a C57BL/6J background (Figure 4B). Both male and female *Crh*^−*120*/+^ mice had significantly increased adiposity from 5 weeks of age (Figure 4, C and D) with a concurrent significant reduction in lean mass (Figure 4, E and 4F). The increased adiposity in the *Crh*^−*120*/+^ mice was associated with dysregulation of glucose (Figure 5, A–D) and lipid metabolism (Figure 5, E and F). Thus, most male and female *Crh*^−*120*/+^ mice had, by the age of 19 weeks, hyperglycemia [plasma glucose > 20 mmol/L (>360 mg/dL)] (Figure 5A) as well as elevated fructosamine concentrations, which indicates a lack of effective glycemic regulation during the preceding weeks (Figure 5B).

These results indicate that 74% of the *Crh*^−*120*/+^ mice had overt diabetes mellitus, although a minority (26%) were observed to have normal concentrations of glucose, indicating the progressive nature of the phenotypes and the variability of the onset of diabetes mellitus associated with CS in these mice. The hyperglycemia in the *Crh*^−*120*/+^ mice was associated with hyperinsulinemia (Figure 5C) and hyperglucagonemia (Figure 5D). Thus, at 8 weeks of age, male and female *Crh*^−*120*/+^ mice had significantly elevated plasma insulin concentrations after a 4-hour fast (Figure 5C), and this hyperinsulinemia persisted at 12 and 16 weeks of age, consistent with likely insulin resistance due to increased adiposity (Figure 4, C and D). Similar elevations in plasma glucagon were also observed in male and female *Crh*^−*120*/+^ mice (Figure 5D). The diabetes mellitus in the *Crh*^−*120*/+^ mice was associated with hypercholesterolemia (Figure 5E) and hypertriglyceridemia (Figure 5F), in 24-week-old mice after a 4-hour fast. The dyslipidemia and adiposity in the *Crh*^−*120*/+^ mice were associated with significantly elevated plasma leptin concentrations, at 8, 12, and 16 weeks of age in male and female *Crh*^−*120*/+^ after a 4-hour fast (Figure 5G). However, plasma adiponectin concentrations were similar in male and female *Crh*^−*120*/+^ mice at 8, 12, and 16 weeks of age after a 4-hour fast (Figure 5H).

### Abnormalities of renal and hepatic function

**Figure 6.**
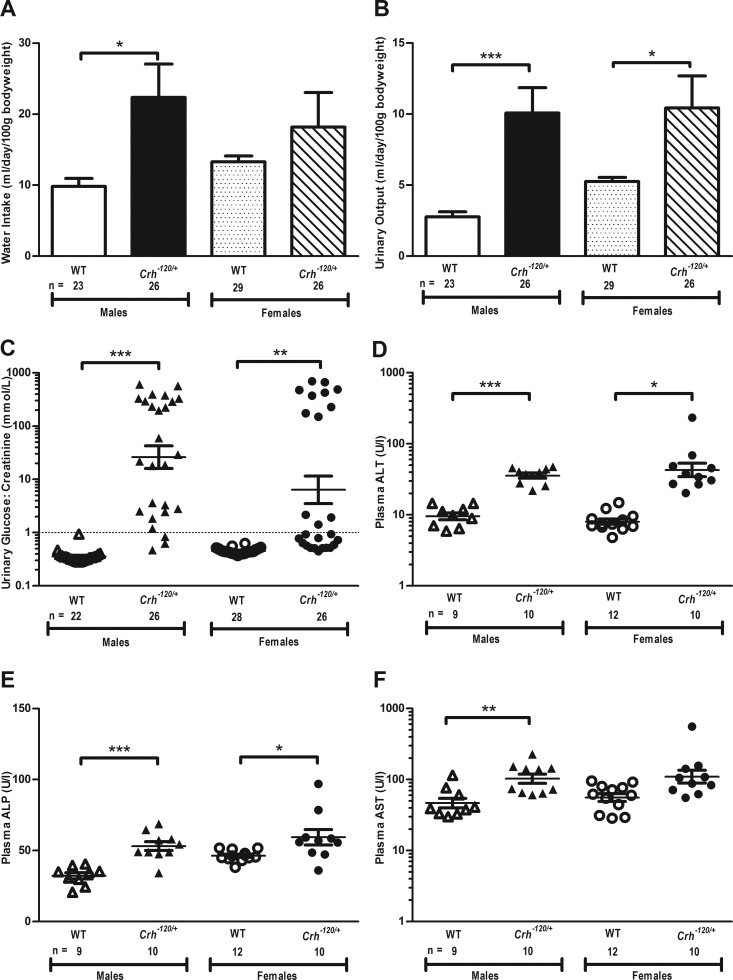
*Crh*^−*120*/+^ mice have impaired renal and hepatic function. A, Water intake measured in metabolic cages over a 24-hour period in WT and *Crh*^−*120*/+^ mice at 12 weeks of age. B, Urinary output measured in metabolic cages over a 24-hour period, in WT and *Crh*^−*120*/+^ mice at 12 weeks of age. C, Urinary glucose to creatinine ratio in WT and *Crh*^−*120*/+^ mice at 12 weeks of age. Plasma ALT (D), plasma ALP (E), and plasma AST activity (F) in 4-hour fasted WT and *Crh*^−*120*/+^ mice between 23 and 27 weeks of age. Results are mean ± SEM, and *P* values were calculated between age- and sex-matched groups using an unpaired *t* test with Welch's correction. *, *P* < .05; **, *P* < .01; ***, *P* < .001.

The effects of diabetes mellitus and insulin resistance on renal and hepatic function were assessed. Metabolic cage studies were undertaken to assess water intake and urinary output over 24 hours (Figure 6). These revealed that male *Crh*^−*120*/+^ had polydipsia (Figure 6A); male and female *Crh*^−*120*/+^ mice had polyuria (Figure 6B), and male and female *Crh*^−*120*/+^ mice had glycosuria (Figure 6C). The female *Crh*^−*120*/+^ mice also had proteinuria (mean ± SEM: WT = 0.7 ± 0.05 vs *Crh*^−*120*/+^ = 1.0 ± 0.1 mmol/L, *P* < .01), and proteinuria has been reported in CS patients (25). Analysis of 4-hour fasted plasma samples at 24 weeks revealed no significant differences in sodium or potassium concentrations in male and female *Crh*^−*120*/+^ mice (Supplemental Table 1), but female *Crh*^−*120*/+^ mice may have been dehydrated, as indicated by the increased creatinine, total protein, albumin, and uric acid concentrations (Supplemental Table 1). No abnormalities were noted in renal morphology on microscopic analysis. Liver function tests revealed mildly elevated activities of ALT and total ALP in male and female *Crh*^−*120*/+^ mice (Figure 6, D and E), and elevated activity of AST in male *Crh*^−*120*/+^ mice only (Figure 6F). These findings are consistent with the moderate diffuse microvesicular vacuolation of hepatocytes observed in *Crh*^−*120*/+^ mice. Thus, renal and hepatic function is impaired in the CS mice, which also have glucose intolerance and insulin resistance.

### Studies of calcium and bone metabolism

**Figure 7.**
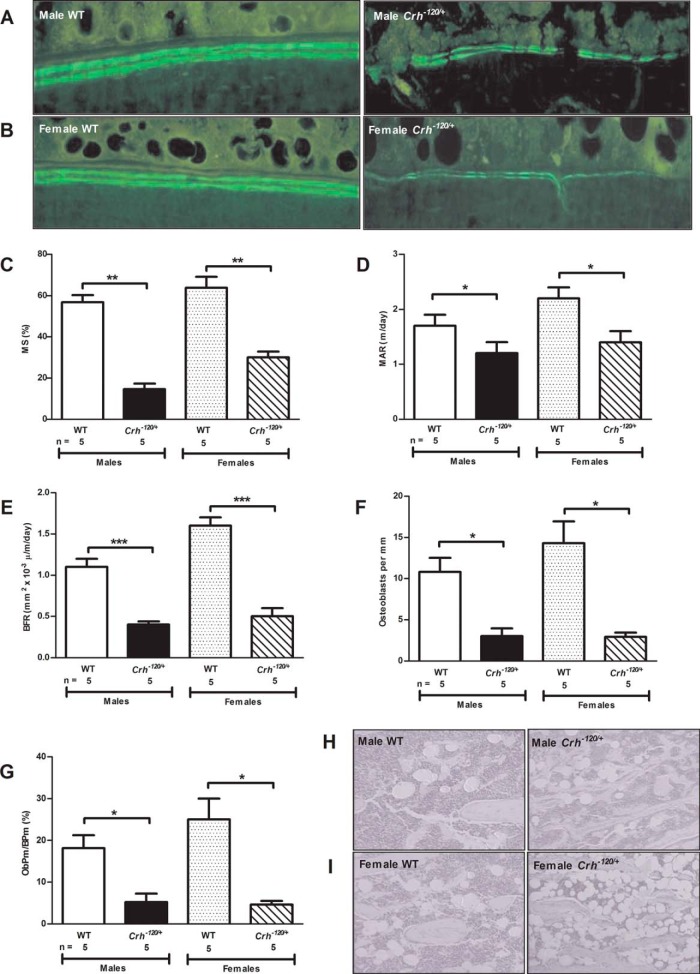
Bone metabolism in adult *Crh*^−*120*/+^ mice aged 12 weeks. A and B, Dynamic histomorphometry of tibia corticoendosteal bone using calcein to label newly formed bone in male *Crh*^−*120*/+^ (A) and female *Crh*^−*120*/+^ (B) mice compared with WT littermates. Mineralizing surface (MS) area (C), MAR (D), bone formation rate (BFR) (E), osteoblast number (F), and percentage of corticoendosteal bone surface covered by osteoblasts (perimeter of the osteoblasts (ObPm)/BPm. in *Crh*^−*120*/+^ and WT littermates (G) are shown. H and I, Adipocytes in the bone marrow of male *Crh*^−*120*/+^ (H) and female *Crh*^−*120*/+^ (I) mice compared with WT littermates. Results are mean ± SEM; *P* values were calculated between age- and sex-matched groups using a two-tailed unpaired Students *t* test. *, *P* < .05; **, *P* < .01; ***, *P* < .001.

The *Crh*^−*120*^ mutation had significant effects on plasma calcium and PTH concentrations, urinary calcium excretion, and rates of bone mineral apposition and formation. Thus, adult male and female *Crh*^−*120*/+^ mice, aged 8–12 weeks, were hypercalcemic (Table 1), and this was associated with significantly reduced plasma PTH concentrations (Table 1), indicating that the hypercalcemia was not due to hyperparathyroidism associated with parathyroid tumors. Plasma phosphate concentrations were not significantly different between *Crh*^−*120*/+^ mice and WT littermates (Table 1). The hypercalcemia was associated with a higher urinary calcium excretion rate (Table 1), consistent with delivery of a higher filtered calcium load to the renal tubules and the decreased renal tubular reabsorption of calcium that would result from the reduced circulating PTH concentrations. The occurrence of hypercalcemia and the increase in total ALP activity (Figure 6E), which may be of hepatic, renal, or bone origin, in the *Crh*^−*120*/+^ suggested an alteration of bone turnover and this was further assessed by measurements of osteocalcin (Table 1) and dynamic histomorphometry (Figure 7). Osteocalcin, whose secretion by osteoblasts, is reduced by glucocorticoids (26, 27), was significantly decreased in plasma of male and female *Crh*^−*120*/+^ mice at 12 weeks of age after a 4-hour fast (Table 1). Moreover, analysis of corticoendosteal bone structure using dynamic histomorphometric studies of undecalcified tibiae collected from mice that were injected with the fluorescent dye calcein to label newly formed bone (Figure 7, A and B) revealed significant reductions in mineralizing surface area (Figure 7C), mineral apposition rates (Figure 7D), and bone formation rates (Figure 7E) in the male and female *Crh*^−*120*/+^ mice. The male and female *Crh*^−*120*/+^ mice also had significant reductions in osteoblast number (Figure 7F) and the percentage of corticoendosteal bone that was covered by osteoblasts (Figure 7G), and this was accompanied by a significant increase in adipocytes in the bone marrow (Figure 7, H and I). Thus, the CS mice have a reduction in osteoblasts that is associated with decreased rates of bone formation and mineral apposition, and this lack of bone calcium deposition may account for the observed hypercalcemia, decreased circulating PTH concentrations, and hypercalciuria.

**Table 1.** Studies of Calcium Metabolism in Adult Mice

	Males^a^		Females^a^	
	WT Mean ± SEM	*Crh*^−*120*/+^ Mean ± SEM	WT Mean ± SEM	*Crh*^−*120*/+^ Mean ± SEM
Calcium, mmol/L^b^	2.28 ± 0.02 (n = 13)	2.40 ± 0.03 (n = 10)^c^	2.32 ± 0.014 (n = 15)	2.45 ± 0.04 (n = 13)^d^
Phosphate, mmol/L^b^	2.48 ± 0.09 (n = 10)	2.59 ± 0.07 (n = 6)	2.47 ± 0.11 (n = 8)	2.47 ± 0.15 (n = 10)
PTH, pmol/L^e^	45.12 ± 6.95 (n = 5)	22.06 ± 3.26 (n = 5)^c^	48.76 ± 7.89 (n = 5)	11.4 ± 1.45 (n = 5)^c^
Calcium to creatinine ratio, mmol/L^f^	0.25 ± 0.03 (n = 22)	0.49 ± 0.05 (n = 24)^g^	0.24 ± 0.01 (n = 28)	0.86 ± 0.13 (n = 25)^g^
Osteocalcin, μg/L^b^	73.48 ± 3.93 (n = 6)	28.37 ± 1.63 (n = 5)^c^	104.13^h^89.12^h^118.82^h^	42.82 ± 2.76 (n = 5)

a Eight- to 12-week-old WT and *Crh*^−*120*/+^ mice were studied.

b Plasma concentrations after a 4-hour fast. Plasma calcium concentrations adjusted for variations in albumin concentrations are shown.

c *P* < .01, calculated between age- and sex-matched groups using a Mann-Whitney test.

d *P* < .05, calculated between age- and sex-matched groups using a Mann-Whitney test.

e Plasma concentrations in free-fed mice.

f Urinary calcium to creatinine ratio.

g *P* < .001, calculated between age- and sex-matched groups using a Mann-Whitney test.

h Plasma osteocalcin was measured in three female WT mice, and the value for each individual mouse, which is provided in the table, is greater than 7 SD above the mean of the female *Crh*^−*120*/+^ mice.

## Discussion

Our studies have established a mouse model for endogenous glucocorticoid excess that is due to a gain-of-function mutation in the promoter region of the *Crh* gene (*Crh-120*) (Figure 3) and thus differs in cause from typical ACTH-dependent CS. Endogenous causes of CS are also rare in patients. The *Crh*^−*120*/+^ mice exhibited some diurnal variation of corticosterone (Figure 1, F and G; Supplemental Figure 1), thereby indicating that the mutant *Crh* promoter remained partially responsive to diurnal alterations in physiological activity. Because the mutant *Crh* promoter is partially responsive to negative feedback, this may explain why plasma ACTH concentrations are lower than might be expected for a model of ACTH-driven glucocorticoid excess. The mutant mice have many of the clinical features of CS due to GC excess and demonstrate the diverse physiological effects exerted by glucocorticoids, which play important roles in protein, lipid, glucose, and bone metabolism (28–30). Thus, increased circulating concentrations of GCs cause an increase in body fat mass specifically by enhancing fat deposition in the visceral compartment and reducing peripheral stores (31); GCs also reduce skeletal muscle mass by decreasing the rate of protein synthesis and by increasing the rate of protein breakdown and increase whole-body lipolysis, resulting in dyslipidemia (32).

The *Crh*^−*120*/+^ mice, which had hypercorticosteronemia (Figure 1), developed many of these features, which included obesity, muscle wasting (Figure 4), and elevated plasma concentrations of cholesterol and triglycerides (Figure 5); the latter is also impacted by impaired hepatic lipid metabolism (Figure 6). Excess GC effects on the liver also contribute to increased hepatic glucose output and hepatic insulin resistance, which in combination with reduced insulin sensitivity in skeletal muscle, results in hyperinsulinemia and hyperglycemia (30). The multiple hormonal imbalances observed in the *Crh*^−*120*/+^ mice can be attributed to both the direct and indirect effects of excess GCs in adipose tissue, skeletal muscle, liver, and pancreatic β-cells, which lead to increased plasma concentrations of leptin (Figure 5), reflecting increased fat mass; hyperinsulinemia indicating insulin resistance; and dysregulation of the counterregulatory hormone glucagon, which exacerbates the hyperglycemia. *Crh*^−*120*/+^ mice when compared with WT littermates did not have significant differences in plasma adiponectin concentrations (Figure 5), a hormone that increases insulin sensitivity in key glucose using tissues. The severity of diabetes mellitus in the *Crh*^−*120*/+^ mice is clearly evident from the level of polydipsia, polyuria, and glycosuria (Figure 6). These abnormalities of glucose and lipid metabolism in the *Crh*^−*120*/+^ mice are similar to those reported in the *Crh* transgenic mouse model, and these include obesity, muscle wasting, hair loss, thin skin, and hypercorticosteronemia (13).

Of note, the *Crh*^−*120*/+^ mice were not hyperpigmented (examined on a C3H/HeH genetic background), despite the presence of increased pituitary *Pomc* expression (Figure 2F). The absence of hyperpigmentation is in keeping with other reported ACTH-driven Cushing's mouse models such as mice transplanted with ACTH-producing pituitary tumors (33) or in the polyoma large T antigen transgenic mice that develop ACTH-producing tumors (34, 35). We postulate that although *Pomc* expression was elevated in CS mice, the synthesized POMC peptide may not have undergone complete posttranslational processing to produce peptides that promote pigmentation, such as α-MSH.

The most notable changes in the adrenal glands were increased weight and hypertrophy of the zona fasciculate. Interestingly, it has been observed in a number of mainly in vitro studies that chronic ACTH stimulation leads to adrenal glomerulosa cells switching their phenotype to fasciculate cells secreting cortisol, rather than aldosterone, and thus resulting in decreased rather than increased secretion of aldosterone (36–38). We did not observe any changes in the zona glomerulosa, although it would be interesting in future studies to examine the expression and secretion of aldosterone in this model.

Some sexual dimorphism was observed in the *Crh*^−*120*/+^ mice with a significant increase in body weight detected only in the female *Crh*^−*120*/+^ mice, due to an increased fat mass from an early age, when compared with male *Crh*^−*120*/+^ mice (Figure 4). Gender-specific differences have also been reported in conditional CRH-overexpressing mice (39) and may be due in part to differences in circulating concentrations of gonadal steroids, which have been shown to influence the hypothalamic-pituitary-adrenal axis (40).

The *Crh*^−*120*/+^ mice also had glucocorticoid-induced osteoporosis (Figure 1), which in man is characterized by rapid bone loss and increased fracture risk within the first 3–6 months of exposure to GC excess, which may be endogenous or exogenous, ie, GC treatment (41). GCs have direct effects on bone cells as well as indirect effects on calcium metabolism, hormone production, and the neuromuscular system (29, 42–44). Chronic exposure to excess GCs suppresses bone formation by osteoblasts and bone resorption by osteoclasts (29), and this in conjunction with increased apoptosis of osteoblasts and osteocytes results in reduced bone quality and bone mass (45, 46). The *Crh*^−*120*/+^ mice illustrated this by having a low BMD (Figure 1), hypercalcemia, hypercalcuria, and decreased plasma concentrations of PTH and osteocalcin (Table 1). The decrease in plasma PTH concentrations is somewhat atypical for Cushing's syndrome because patients with this frequently have secondary hyperparathyroidism (47, 48). In the *Crh*^−*120*/+^ mice, it is likely that the hypercalcemia resulted in a suppression of PTH secretion.

The *Crh*^−*120*/+^ mice also had reductions in the bone mineralizing surface area, mineral apposition rate, bone formation rate, osteoblast number and corticoendosteal bone covered in osteoblasts (Figure 7). Moreover, the reduction in osteoblast number in the bone marrow of *Crh*^−*120*/+^ mice was associated with an increase in adipocytes, suggesting a preferential differentiation of bone marrow stromal cells from osteoblastic to adipocyte lineage, consistent with steroid-induced osteoporosis (49). Together these findings indicate a reduction in bone quality and provide evidence of osteoporosis, making this an important mouse model for the study of the development of osteoporosis and therapeutic interventions. A number of in vivo models for GC excess have been published (Supplemental Table 2) with a range of reported phenotypes including metabolic, behavioral, motor function, hair, and pigmentation anomalies. However, there is a paucity of such models for glucocorticoid-induced osteoporosis, and other reported rodent models require either ovariectomy or immobilization to induce bone loss (50). A mouse model for exogenous GC-induced osteoporosis has been previously developed and successfully used to provide mechanistic insights into this disorder and also to evaluate osteoporosis therapies (46, 51, 52). In contrast, the *Crh*^−*120*/+^ mice provide a model of GC-induced osteoporosis that is due to endogenous GC excess, and this may be of use for characterizing the longer-term effects of GC on bone. Of note, CRH-overexpressing transgenic mice have been recently reported to have a low bone mass phenotype consistent with our observations on the *Crh*^−*120*/+^ mice (16). Thus, the *Crh*^−*120*/+^ mouse model that develops steroid-induced osteoporosis represents an important advance for this common disorder that results in significant morbidity and mortality.

The T-to-C mutation at −120 of the mouse *Crh* promoter, which involves a highly evolutionary conserved nucleotide, represents an important but rare example of a gain of function involving a promoter region (Figure 3) (21). Vertebrate CRH promoter regions are very highly conserved with human, rat, and ovine promoters having 94% similarity greater than 330 bp (21), and regions of up to 75% homology extend for several kilobases beyond this in mammalian species (http://ecrbrowser.dcode.org) (53). Moreover, using the transcriptional element search system to search the region of DNA containing the −120-bp mutation (Figure 3) identified two putative transcription factor binding sites, spanning the mutated base, for *Cdx-1*/*Cad* and EF11 (54). Caudal-type homeobox 1 (CDX1), whose transcription is involved in regulating *Hox* genes and is linked to the development of the axial skeleton as well as a likely involvement in development of the female urogenital system, is regulated by retinoic acid (55–58). Any use of this potential transcription factor binding site by CDX1 in the context of the *Crh* promoter remains unknown. However, studies have indicated that this element forms part of a second independent cAMP response element, CDXARE, that escapes inhibition by a negative GC response element within the promoter and is stimulated by glucocorticoids and cAMP (22, 23). The T-to-C mutation of the *Crh*^−*120*/+^ mice resulted in an increase in promoter transcription in the reporter assay (Figure 3D), thereby indicating that the mutation either prevented inhibition through this element or acted positively to enhance transcription factor binding and activity; either possibility would result in increased transcription and thus effectively a gain-of-function mutation.

In summary, we report the identification of a genetic mouse model with the features of CS in an ENU screen for new mouse models of metabolic disorders. This model was initially identified as an obese, diabetic mouse that had osteoporosis. Mapping of the underlying mutation identified a point mutation at −120 of the *Crh* gene promoter, and functional in vitro characterization showed that this mutation increased CRH expression. Thus, a novel gain-of-function promoter mutation of CRH has been identified, and this provides insights into the regulation of this gene and raises the possibility that *CRH* promoter mutations may be a cause of CS in man. The *Crh*^−*120*/+^ mouse model also represents a valuable resource for investigating the pathophysiology of GC excess and steroid-induced osteoporosis.
